# Electroneurographic profiles of peripheral neuropathy in diabetic patients in Lubumbashi

**DOI:** 10.3389/fneur.2026.1928343

**Published:** 2026-09-23

**Authors:** Jean-Paul Ngoy Mande, Eunice Bilonda Mbuyamba, Paul Musa Obadia, Dophra Ngoy Nkulu, Célestin Banza Lubaba, Abdon Sylvestre Mukalay wa Mukalay, Christophe Lelubre, Laurence Ris, Daniel Okitundu Luwa E-Andjafono

**Affiliations:** 1 Department of Neurology and Psychiatry, Faculty of Medicine, University of Lubumbashi, Lubumbashi, Democratic Republic of Congo; 2 Toxicology and Environment Unit, Department of Public Health, University of Lubumbashi, Lubumbashi, Democratic Republic of Congo; 3 Lubumbashi Cardiology Center, Lubumbashi, Democratic Republic of Congo; 4 Department of Internal Medicine, Faculty of Medicine, University of Lubumbashi, Lubumbashi, Democratic Republic of Congo; 5 Unit of Clinical Epidemiology and Tropical Disease, Department of Public Health, University of Lubumbashi, Lubumbashi, Democratic Republic of Congo; 6 Neuroscience Department, Faculty of Medicine and Pharmacy, University of Mons, Mons, Belgium; 7 Department of Neurology, Neuropsychopathological Center, Faculty of Medicine, University of Kinshasa, Kinshasa, Democratic Republic of Congo

**Keywords:** diabetes mellitus, neuropathic pain, electroneurography, peripheral neuropathy, Lubumbashi, DR.Congo

## Abstract

**Introduction:**

Electroneurography (ENG) provides an objective tool for assessing nerve function and characterizing electrophysiological profiles. The underutilization of ENG in our setting limits the early diagnosis of peripheral neuropathy (PN), a frequent complication of type 2 diabetes. This study aimed to describe electroneurographic parameters in diabetic subjects (DS) in relation to neuropathic pain (NP) and to compare them with non-diabetic controls (NDS) in Lubumbashi, in order to identify abnormalities specifically attributable to diabetes.

**Methods:**

A cross-sectional study included 51 participants (mean age 49.7 ± 11.8 years): 25 DS and 26 NDS. NP was assessed using the validated DN4 questionnaire. ENG recordings were performed with a Neuro-MEP Micro device, following standard protocols for sensory and motor nerve exploration.

**Results:**

NP was significantly more frequent in DS (68%) than in NDS (46%), with a peak in insulin-dependent DS (90%; *p* < 0.05). All ENG parameters—amplitude, distal latency, and conduction velocity—were significantly altered in DS (*p* < 0.05), indicating a predominantly axonal, sensory-motor polyneuropathy with demyelinating features, mainly affecting the lower limbs. Patient-level analysis showed that diabetic patients with abnormal ENG had longer diabetes duration (*p* = 0.04), higher HbA1c (*p* = 0.02), and more frequent NP (*p* = 0.03). ENG abnormalities not associated with NP were observed, suggesting subclinical neuropathies.

**Conclusion:**

Combining ENG with the DN4 score facilitates the characterization of diabetic peripheral neuropathy (DPN) and enables the detection of subclinical involvement. Integrating ENG into routine clinical practice in Lubumbashi is essential for early diagnosis. Further studies are needed to establish local normative values and refine electroneurographic profiles.

## 1 Introduction

Diabetes mellitus is now recognized as one of the most serious and prevalent chronic diseases worldwide (1). It is among the four priority non-communicable diseases identified by the World Health Organization (2). As a veritable metabolic pandemic, its prevalence has nearly doubled over the past four decades, increasing from 4.7% in 1980 to 8.5% in the adult population (3). This increase affects both high-income countries and those with low- or middle-income economies.

In the Democratic Republic of Congo (DRC), diabetes mellitus represents a growing public health concern. Available data indicate a prevalence ranging from 1.7 to 18%, with particularly high rates observed in urban areas (4–6). This heterogeneity reflects variations in access to diagnosis, dietary habits, sedentary lifestyles, and rapid urbanization. The precise prevalence of diabetes in Lubumbashi remains poorly defined; however, a study conducted at the University Clinics of Lubumbashi reported a prevalence of 14% among patients hospitalized in internal medicine.

The circumstances surrounding the diagnosis of diabetes vary depending on the setting (7), ranging from typical clinical manifestations to acute and chronic complications. Among these complications, peripheral neuropathy (PN), specifically diabetic peripheral neuropathy (DPN), is one of the most frequent and debilitating, contributing to significant morbidity and impaired quality of life.

Diabetic peripheral neuropathy encompasses a heterogeneous group of disorders affecting the peripheral nervous system (PNS), distinguished by their anatomical characteristics, clinical courses, and diverse phenotypes (8). It can lead to irreversible complications, including sensory loss, postural instability, falls, foot ulcers, amputations, and a significant deterioration in quality of life (9).

The most common type of nerve damage is axonal or demyelinating, typically symmetric and distal, predominantly affecting the lower limbs, with a distal-to-proximal severity gradient (10, 11). Distal symmetric polyneuropathy represents the most frequent clinical presentation of DPN (12). It often manifests as neuropathic pain characterized by sensations of burning, painful cold, electric shocks, tingling, paresthesias, numbness, or itching in the extremities (13).

DPN may be present at the time of diagnosis of type 2 diabetes (14). Its prevalence varies depending on the diagnostic criteria and tools used; based on clinical examination alone, estimates range from 30 to 66% (13, 15). The underlying pathophysiological mechanisms remain complex and incompletely understood (16). In Lubumbashi, no studies have reported the prevalence of this chronic complication of diabetes. However, diabetic ketoacidosis has been observed in 5% of patients (17) and diabetic retinopathy in 13% (18).

Metabolic, vascular, genetic, and, more recently, inflammatory factors appear to interact synergistically in the pathogenesis of this chronic complication (19). Additional susceptibility factors, such as age, sex, height, alcohol abuse, and nutritional deficiencies, may also influence its development.

Often progressing insidiously, DPN frequently remains undiagnosed until severe complications arise, most notably diabetic foot, recognized by the WHO as a common and serious issue in resource-limited settings. Early detection is therefore essential to prevent these irreversible outcomes.

Sensory and motor nerve conduction studies, performed using electroneurography (ENG), enable objective assessment of DPN and confirmation of the diagnosis based on electrophysiological abnormalities correlated with clinical signs and symptoms (20). ENG is a non-invasive reference standard for detecting neuropathies, including subclinical forms (21). It allows for the differentiation between axonal and demyelinating involvement, providing valuable prognostic and therapeutic information.

However, ENG remains underutilized in the Democratic Republic of Congo, particularly in Lubumbashi, where it has not yet been systematically integrated into the diagnostic pathway.

This exploratory study aimed to characterize neuropathic pain and analyze motor and sensory nerve conduction parameters in diabetic subjects compared to non-diabetic subjects, with the goal of contributing to the implementation of early screening strategies, both clinical and electrophysiological, for diabetic neuropathy in our setting.

## 2 Methods

### 2.1 Study setting

The study was conducted in Lubumbashi, the capital city of Haut-Katanga Province (Democratic Republic of Congo), which has an estimated population of 1.3 million inhabitants and covers an area of 747 km^2^.

### 2.2 Study design

This was a multi-center, cross-sectional case–control study conducted from September 2018 to December 2019, involving diabetic and non-diabetic subjects recruited from multiple healthcare facilities in Lubumbashi.

### 2.3 Study population and sampling

The study population consisted of diabetic patients followed at or presenting for the first time to the following facilities: University Clinics of Lubumbashi, Jason Sendwe General Referral Hospital, Gecamines South Hospital, and Shalina Medical Center.

Inclusion criteria for the diabetic group:

- Age between 18 and 64 years
- Confirmed diagnosis of type 2 diabetes
- Residency in Lubumbashi for at least 3 years
- Provision of informed consent to participate in the study

#### 2.3.1 Control group

The control group consisted of non-diabetic subjects presenting to the same facilities during the same period for acute non-metabolic conditions (benign infections, minor trauma, routine check-ups, non-specific musculoskeletal pain), with no history of diabetes or known metabolic disorders. They were matched with diabetic subjects for age, sex, and duration of residency in Lubumbashi.

#### 2.3.2 Exclusion criteria

Subjects with incomplete data, significant edema, severe obesity, persistent cold extremities despite warming, coagulation disorders, a pacemaker, or a metallic joint prosthesis were excluded.

1. In total, a sample of 51 participants was recruited, comprising 25 type 2 diabetic subjects and 26 non-diabetic subjects forming the comparison group. The sample size of 22 cases was estimated based on a correlation of 0.2, a probability of exposed controls of 46%, an odds ratio of 9, a control-to-case ratio of 1, an alpha level of 0.05, and a power of 80% (22); StatsDirect Statistical Software. (2024). Version 4.0.4. Available at: https://www.statsdirect.com/.

### 2.4 Data collection tools and procedures

Sociodemographic and clinical data were collected using a standardized form that included information on personal and family medical history. Neuropathic pain was assessed using the DN4 (Neuropathic Pain in 4 Questions) questionnaire, which has a sensitivity of 83% and a specificity of 90% (23). The DN4 comprises 10 items divided into four questions:

- Seven items addressing the description of pain (burning, painful cold, electric shocks, tingling, pins and needles, numbness, itching)
- Three items addressing the neurological examination (hypoesthesia to touch, hypoesthesia to pinprick, tactile allodynia)

Each positive item receives a score of 1, and a negative item a score of 0, for a total range of 0 to 10. A score ≥ 4 defines the presence of neuropathic pain.

Blood samples were collected at the laboratory of the University Clinics of Lubumbashi by a trained technician.

- Glycated hemoglobin (HbA1c) was measured by immunofluorescence on EDTA tubes.
- Glucose was measured by the glucose-oxidase enzymatic method.
- Urea was measured by the urease enzymatic kinetic method.
- Creatinine was measured using the Jaffé technique.

### 2.5 Electroneurographic examinations

Electroneurography was performed using the Neuro-MEP Micro device (Neurosoft, France, version 2009). All examinations were conducted in a temperature-controlled room (ambient temperature ≥ 24 °C). Limb temperature was monitored using a skin thermometer.

Motor nerve conduction studies were performed using standard surface stimulation and recording techniques. Sensory nerve conduction studies were performed using an antidromic technique, with surface electrodes (22 × 30 mm) for the median, ulnar, and radial nerves, and needle electrodes (10 × 0.30 mm) for the superficial peroneal and sural nerves. Peak latencies were measured for sensory responses. Stimulation and recording sites followed standard anatomical landmarks (24).

The reference values used for classification of abnormalities were those provided by the device manufacturer (Neuro-MEP Micro), which are consistent with European consensus standards (24, 25) and presented in Table 1. An abnormality was defined as a value below the lower limit of normal (or above the upper limit for distal latencies) based on these reference values.

**Table 1:** Reference values for motor and sensory nerve conduction studies according to European standards.

Nerve	Modality	Conduction velocity (m/s)	Amplitude	Distal latency (ms)
Median	Motor	> 50	> 6 mV	< 3.7
Median	Sensory	> 50	> 15 μV	–
Ulnar	Motor	> 50	> 6 mV	< 3.2
Ulnar	Sensory	> 50	> 10 μV	–
Radial	Motor	> 53	> 6 mV	< 3.4
Radial	Sensory	> 50	> 15 μV	–
EPS (Peroneal)	Motor	> 42	> 3 mV	< 5.0
EPS (Superficial peroneal)	Sensory	> 40	> 10 μV	–
IPS (Posterior tibial)	Motor	> 42	> 6 mV	< 5.5
IPS (Sural)	Sensory	> 40	> 10 μV	–

EPS, external popliteal sciatic nerve (common peroneal nerve for motor, superficial peroneal nerve for sensory); IPS, internal popliteal sciatic nerve (tibial nerve for motor, sural nerve for sensory). Sensory amplitudes are expressed in microvolts (μV); motor amplitudes in millivolts (mV). Distal motor latency is expressed in milliseconds and motor or sensory nerve conduction velocity in meters per second (m/s).

### 2.6 Electrophysiological classification criteria

Electrophysiological patterns were classified according to the following criteria, adapted from established guidelines (20, 24):

- *Distal symmetric polyneuropathy*: Bilateral, symmetric reduction of amplitudes and/or conduction velocities in the lower limbs, with a distal-to-proximal gradient, affecting both motor and sensory nerves.
- *Focal neuropathy*: Unilateral abnormality in a single nerve territory, with normal findings in other nerves.
- *Multifocal neuropathy*: Asymmetric abnormalities affecting two or more non-contiguous nerves.
- *Mixed axonal-demyelinating neuropathy*: Co-existence of reduced amplitudes (axonal) and prolonged distal latencies or reduced conduction velocities (demyelinating) in the same nerve.
- *Axonal neuropathy*: Predominant reduction of amplitudes with normal or mildly reduced conduction velocities and normal distal latencies.
- *Demyelinating neuropathy*: Predominant prolongation of distal latencies and/or reduction of conduction velocities with normal or mildly reduced amplitudes.

These classifications were applied at the patient level based on the overall electroneurographic pattern, taking into account all tested nerves.

### 2.7 Statistical analysis

Data were encoded using Epi Info 7.0 and analyzed with Excel 2019 and SPSS version 24. Quantitative variables were expressed as mean ± standard deviation or median (Q1–Q3) depending on the distribution, assessed using the Shapiro–Wilk or Anderson-Darling test. Qualitative variables were presented as frequencies and percentages.

Mean comparisons used Student’s t-tests (parametric) or Mann–Whitney/Wilcoxon tests (non-parametric). Proportions were compared using *χ*^2^ or Fisher’s exact test. Associations between categorical variables were assessed using odds ratios (OR) and their 95% confidence intervals. Correlations were calculated using Pearson or Spearman coefficients as appropriate. A *p*-value < 0.05 was considered statistically significant.

### 2.8 Ethical considerations

This study received approval from the Ethics Committee of the University of Lubumbashi under reference number UNILU/CEM/150/2019.

Data collection was conducted in accordance with the ethical principles outlined in the Declaration of Helsinki for medical research involving human subjects.

Free, informed, and written consent was obtained from each participant prior to enrollment.

Anonymity, confidentiality of information, and respect for the privacy of participants were rigorously guaranteed throughout the research process.

## 3 Results

### 3.1 General characteristics of participants

The sociodemographic and clinical characteristics of diabetic subjects (DS) and non-diabetic subjects (NDS) are presented in Table 2. No significant differences were observed between the two groups for demographic parameters (age, sex, height, weight, abdominal perimeter, blood pressure, body mass index, or duration of residency in Lubumbashi) (*p* > 0.05 for all comparisons).

**Table 2:** Demographic, clinical, biological, and neuropathic pain characteristics of study participants.

Characteristic	All subjects (*n* = 51)	Non-diabetics (*n* = 26)	Diabetics (*n* = 25)	*p*-value
Gender, *n* (%)				>0.05
Male	28 (54.9)	13 (50.0)	15 (60.0)	–
Female	23 (45.1)	13 (50.0)	10 (40.0)	–
Median age, years (P25–P75)	53 (41–59)	45 (39–55)	55 (50–59)	0.07
Median height, cm (P25–P75)	165 (161–170)	165 (160–170)	165 (162–175)	0.3
Median weight, kg (P25–P75)	70 (62–78)	68 (60–78)	71 (63–78)	0.5
Median SBP, mmHg (P25–P75) *	124 (113–132)	124 (113–133)	124 (111–131)	0.5
Median DBP, mmHg (P25–P75) *	78 (70–84)	78 (74–86)	76 (68–84)	0.3
Median BMI, kg/m^2^ (P25–P75)	25 (22–28)	26 (22–28)	25 (23–27)	0.5
Median ABP, cm (P25–P75)	92 (81–97)	90 (80–97)	92 (82–96)	0.8
Median TP, cm (P25–P75)	94 (87–98)	94 (87–98)	94 (90–97)	>0.9
Median LTL, years (P25–P75)	30 (17–49)	26 (17–46)	36 (11–49)	0.7
Biochemical characteristics				
Blood glucose, mg/dL (P25–P75)	104 (88–181)	88 (83–95)	181 (155–264)	<**0.001**
HbA1c, % (P25–P75)	5.94 (4.95–7.00)	5.53 (4.81–6.07)	6.85 (5.70–7.46)	**0.006**
Urea, mg% (P25–P75)	27 (22–32)	24 (20–30)	29 (24–36)	**0.040**
Creatinine, mg% (P25–P75)	1.10 (0.99–1.31)	1.11 (1.02–1.27)	1.06 (0.97–1.32)	0.9
Neuropathic pain symptoms (DN4), *n* (%)				
Burning		15 (57.7)	20 (80.0)	0.13
Painful cold		8 (30.7)	8 (32.0)	1.0
Electrical shocks		13 (50.0)	13 (52.0)	1.0
Tingling		15 (57.7)	20 (80.0)	0.13
Pins and needles		12 (46.1)	18 (72.0)	0.08
Numbness		12 (46.1)	14 (56.0)	0.57
Itching		6 (23.1)	13 (52.0)	**0.04**
Hypoesthesia to touch		7 (26.9)	8 (32.0)	0.76
Hypoesthesia to pinprick		2 (7.7)	3 (12.0)	0.66
Brushing		4 (15.3)	4 (16.0)	1.0

ABP, abdominal perimeter; BMI, body mass index; DBP, diastolic blood pressure; DN4, neuropathic pain in 4 questions; HbA1c, glycated hemoglobin; LTL, length of time lived (duration of diabetes in diabetic group); SBP, systolic blood pressure; TP, thigh perimeter. Data are presented as median (P25–P75) or *n* (%). *p*-values in bold indicate statistically significant differences between groups (*p* < 0.05). *n* = 50 for blood pressure measurements.

In contrast, glucose, glycated hemoglobin (HbA1c), and urea levels were significantly higher in DS compared to NDS (*p* < 0.05) (Table 2). These differences confirm the validity of the metabolic distinction between the two groups.

Significant differences between diabetic and non-diabetic groups are also presented in Table 3 for electroneurographic parameters (amplitudes, distal latencies, and conduction velocities).

**Table 3:** Electroneurographic parameters in non-diabetic and diabetic subjects.

Nerve	Side	Parameter	Standard	Non-diabetics (*n* = 26)	Diabetics (*n* = 25)	*p*-value	Type of lesion
Median							
Motor	R	Amplitude (mV)	> 6	9.72 (7.73–11.30)	8.00 (6.17–9.61)	**0.034**	–
	R	Distal latency (ms)	< 3.7	4.21 (4.02–4.35)	4.68 (4.18–5.07)	**0.008**	Myelinopathy
	R	Velocity (m/s)	> 50	54.1 (51.4–56.3)	51.3 (50.2–56.0)	0.3	–
	L	Amplitude (mV)	> 6	9.3 (8.0–13.1)	8.5 (6.9–10.0)	**0.028**	–
	L	Distal latency (ms)	< 3.7	4.08 (3.90–4.33)	4.68 (4.16–5.08)	**0.001**	Myelinopathy
	L	Velocity (m/s)	> 50	55.1 (52.5–58.1)	50.2 (44.2–55.1)	**0.005**	Myelinopathy
Sensory	R	Amplitude (μV)	> 15	27 (15–32)	13 (6–24)	**0.004**	Axonopathy
	R	Distal latency (ms)	–	2.53 (2.34–2.91)	2.76 (2.54–3.28)	0.085	–
	R	Velocity (m/s)	> 50	58 (52–64)	54 (45–60)	0.072	–
	L	Amplitude (μV)	> 15	23 (16–31)	12 (8–19)	<**0.001**	Axonopathy
	L	Distal latency (ms)	–	2.57 (2.37–2.75)	2.41 (1.57–3.08)	0.7	–
	L	Velocity (m/s)	> 50	58 (54–61)	62 (49–82)	0.6	–
Ulnar							
Motor	R	Amplitude (mV)	> 6	8.48 (6.86–9.65)	7.11 (6.12–7.94)	**0.018**	–
	R	Distal latency (ms)	< 3.2	4.24 (3.95–4.54)	4.72 (4.38–5.10)	**0.004**	Myelinopathy
	R	Velocity (m/s)	> 50	55.2 (51.4–58.8)	50.4 (47.6–55.2)	**0.017**	Myelinopathy
	L	Amplitude (mV)	> 6	7.90 (7.10–9.45)	7.88 (5.45–8.80)	0.3	–
	L	Distal latency (ms)	< 3.2	4.04 (3.82–4.46)	4.74 (4.36–4.98)	<**0.001**	Myelinopathy
	L	Velocity (m/s)	> 50	57 (53–59)	53 (50–53)	**0.002**	Myelinopathy
Sensory	R	Amplitude (μV)	> 10	11 (7–22)	10 (6–15)	0.5	–
	R	Distal latency (ms)	–	2.30 (2.14–2.47)	2.32 (1.92–2.52)	>0.9	–
	R	Velocity (m/s)	> 50	59 (53–64)	59 (54–74)	0.6	–
	L	Amplitude (μV)	> 10	13.8 (11.0–17.9)	6.6 (4.5–14.0)	**0.008**	Axonopathy
	L	Distal latency (ms)	–	2.29 (2.00–2.46)	2.40 (2.04–2.54)	0.5	–
	L	Velocity (m/s)	> 50	59 (55–69)	56 (55–62)	0.8	–
Radial							
Motor	R	Amplitude (mV)	> 6	6.15 (4.89–6.71)	5.09 (3.73–5.86)	**0.047**	Axonopathy
	R	Distal latency (ms)	< 3.4	3.09 (2.77–3.29)	3.42 (3.10–3.88)	**0.010**	Myelinopathy
	R	Velocity (m/s)	> 53	55.6 (53.3–58.0)	50.0 (48.2–52.3)	<**0.001**	Myelinopathy
	L	Amplitude (mV)	> 6	6.16 (4.72–7.18)	4.70 (4.14–5.24)	**0.023**	Axonopathy
	L	Distal latency (ms)	< 3.4	3.09 (2.92–3.38)	3.50 (3.02–3.82)	**0.012**	Myelinopathy
	L	Velocity (m/s)	> 53	57 (52–61)	52 (48–55)	**0.003**	Myelinopathy
Sensory	R	Amplitude (μV)	> 15	26 (13–36)	15 (9–26)	**0.007**	Axonopathy
	R	Distal latency (ms)	–	0.93 (0.81–1.20)	1.23 (0.94–1.56)	**0.021**	–
	R	Velocity (m/s)	> 50	68 (62–78)	66 (55–75)	0.3	–
	L	Amplitude (μV)	> 15	25 (16–34)	15 (11–20)	**0.003**	Axonopathy
	L	Distal latency (ms)	–	0.91 (0.74–1.02)	1.36 (1.12–1.60)	<**0.001**	–
	L	Velocity (m/s)	> 50	77 (65–85)	63 (58–70)	**0.004**	–
External popliteal sciatic (EPS)							
Motor	R	Amplitude (mV)	> 3	5.33 (4.79–7.26)	2.85 (1.59–4.76)	<**0.001**	Axonopathy
	R	Distal latency (ms)	< 5.0	6.46 (6.04–7.21)	7.76 (6.80–8.64)	<**0.001**	Myelinopathy
	R	Velocity (m/s)	> 42	46.3 (44.2–50.7)	41.1 (37.0–45.2)	<0.001	–
	L	Amplitude (mV)	> 3	5.77 (3.93–7.38)	3.62 (2.67–5.43)	**0.012**	Axonopathy
	L	Distal latency (ms)	< 5.0	6.82 (6.08–7.04)	8.38 (7.34–8.95)	<**0.001**	Myelinopathy
	L	Velocity (m/s)	> 42	46.7 (44.5–49.5)	40.8 (37.9–45.4)	<**0.001**	Myelinopathy
Sensory	R	Amplitude (μV)	> 10	14 (9–22)	8 (6–12)	**0.008**	Axonopathy
	R	Distal latency (ms)	–	2.39 (1.96–2.63)	2.56 (2.16–3.08)	0.13	–
	R	Velocity (m/s)	> 41	41 (37–46)	47 (42–56)	0.073	–
	L	Amplitude (μV)	> 10	13 (10–19)	9 (3–14)	**0.015**	Axonopathy
	L	Distal latency (ms)	–	2.31 (2.12–2.69)	2.44 (1.98–2.74)	0.7	–
	L	Velocity (m/s)	> 41	48 (43–51)	44 (40–50)	0.3	–
Internal popliteal sciatic (IPS)							
Motor	R	Amplitude (mV)	> 6	6.4 (5.2–9.5)	5.0 (3.0–7.6)	0.10	–
	R	Distal latency (ms)	< 5.5	8.71 (7.80–9.92)	10.20 (9.66–11.90)	<**0.001**	Myelinopathy
	R	Velocity (m/s)	> 42	49 (43–52)	41 (37–44)	<0.001	–
	L	Amplitude (mV)	> 6	6.4 (5.2–9.5)	5.0 (3.0–7.6)	0.10	–
	L	Distal latency (ms)	< 5.5	8.71 (7.80–9.92)	10.20 (9.66–11.90)	<**0.001**	Myelinopathy
	L	Velocity (m/s)	> 42	46 (42–49)	39 (37–43)	**0.001**	–
Sensory	R	Amplitude (μV)	> 10	23 (12–39)	6 (4–14)	<**0.001**	Axonopathy
	R	Distal latency (ms)	–	1.61 (1.42–1.79)	1.74 (1.30–2.12)	0.4	–
	R	Velocity (m/s)	> 41	44 (39–53)	46 (37–54)	0.9	–
	L	Amplitude (μV)	> 10	28 (12–39)	10 (3–15)	<**0.001**	Axonopathy
	L	Distal latency (ms)	–	1.39 (1.19–1.65)	1.80 (1.52–2.10)	**0.011**	–
	L	Velocity (m/s)	> 41	49 (41–59)	44 (40–50)	0.14	–

EPS, external popliteal sciatic nerve (common peroneal nerve); IPS, internal popliteal sciatic nerve (tibial nerve); L, left; R, right. Data are presented as median (P25–P75). Type of lesion is suggested based on the parameter most affected: myelinopathy = prolonged distal latency and/or reduced conduction velocity; axonopathy = reduced amplitude. *p*-values in bold indicate statistically significant differences between groups (*p* < 0.05).

### 3.2 Assessment of neuropathic pain (DN4)

Symptoms of neuropathic pain, as assessed by the DN4 questionnaire, were more frequent in DS than in NDS. The most commonly reported manifestations in DS were burning sensations or pins and needles (80% vs. 57.7%), tingling (72% vs. 46.1%), and numbness (56% vs. 46.1%). Itching was also significantly more frequent in DS (52%) than in NDS (23.1%) (*p* = 0.04).

As shown in Table 4, a pathological DN4 score (≥ 4) was observed in 68% of DS compared to 46% of NDS. The highest proportion was observed in insulin-dependent DS (90%), compared to 46% in NDS (*p* = 0.024). A significant difference was also noted for the raw DN4 score between insulin-dependent DS and NDS (*p* = 0.014). However, the difference between insulin-dependent and non-insulin-dependent DS was not statistically significant (*p* = 0.088).

**Table 4:** DN4 scores and pathological neuropathic pain classification according to diabetic status and insulin dependence.

Compared group	Reference group	*n*	DN4 raw score, median (P25–P75)	DN4 ≥ 4 (pathological), n (%)	*p*-value*
Non-diabetics	–	26	3.00 (2.00–5.00)	12 (46.2)	–
Diabetics (all)	Non-diabetics	25	5.00 (3.00–6.00)	17 (68.0)	0.16
Diabetics with insulin dependence (ID)	Non-diabetics	10	6.00 (5.00–6.00)	9 (90.0)	**0.014**
Diabetics non-ID	–	15	4.00 (2.50–6.00)	8 (53.3)	–
Diabetics ID	Diabetics non-ID	10	6.00 (5.00–6.00)	9 (90.0)	0.088

DN4, Douleur Neuropathique 4 questionnaire (validated screening tool for neuropathic pain); ID, insulin dependence. A DN4 score ≥ 4 is considered pathological (indicative of neuropathic pain). Data are presented as median (P25–P75) or *n* (%). *p*-values in bold indicate statistically significant differences (*p* < 0.05). Statistical tests: Mann–Whitney test for raw scores; *χ*^2^ or Fisher’s exact test for proportions.

### 3.3 Electroneurographic abnormalities

Electroneurographic parameters motor and sensory conduction velocities, amplitudes, and distal latencies were significantly altered in DS compared to NDS (Table 3). Conduction velocities and amplitudes were generally reduced, while distal latencies were prolonged.

Based on the device’s reference values (consistent with European standards), the neuropathic profiles observed in DS included:

1. Bilateral demyelinating motor neuropathy and left axonal motor neuropathy of the common peroneal nerve.
2. Bilateral axonal motor neuropathy of the posterior tibial nerve.
3. Unilateral right demyelinating motor neuropathy and bilateral axonal motor neuropathy of the radial nerve.
4. Bilateral demyelinating motor neuropathy of the ulnar and median nerves.

In NDS, only bilateral demyelinating motor neuropathies of the median and ulnar nerves were noted, characterized by prolonged distal latencies.

Regarding sensory parameters, significant differences in sensory amplitude were observed for the majority of bilateral nerves, with the exception of the right ulnar nerve. Differences in sensory latency were significant for the left tibial nerve and left radial nerve, while sensory velocities were significantly altered only for the left radial nerve. No definite sensory neuropathy was identified based on the device’s normative values.

### 3.4 Association between neuropathic pain (DN4) and electroneurographic parameters

Correlation analyses (Spearman’s test) showed generally negative correlations between the DN4 score and electroneurographic parameters, with the exception of motor conduction velocity and amplitude of the right radial nerve. However, these correlations were not statistically significant, with the exception of the correlation between sensory conduction velocity of the left tibial nerve and the DN4 score (*r* = −0.285; *p* = 0.042).

Overall, no significant association was observed between the presence of neuropathic pain and the frequency of amplitude or velocity abnormalities on ENG for the total population.

However, in diabetic subjects, neuropathic pain was significantly associated with reduced sensory conduction velocity of the left common peroneal nerve [9/17 (52.9%) vs. 0/8 (0%); OR = 1.889 (95% CI: 1.283–3.518)] and the left tibial nerve [8/17 (47.1%) vs. 0/8 (0%); OR = 1.889 (95% CI: 1.207–2.957)]. A significant negative association was also noted between neuropathic pain and reduced motor conduction velocity of the right radial nerve [5/17 (29.4%) vs. 7/8 (87.5%); OR = 0.06 (95% CI: 0.006–0.618)].

In non-diabetic subjects, no significant correlations were observed for either sensory or motor conduction.

In the upper limbs, reduced sensory conduction velocity was significantly associated with diabetes for the right median nerve [11.5% vs. 44%; OR = 6.024 (95% CI: 1.428–25.403)], while no other associations were found. For motor conduction velocity, the significant association was only for the right radial nerve [11.5% vs. 48%; OR = 7.077 (95% CI: 1.683–29.761)].

The risk of decreased nerve conduction amplitudes in the common peroneal and tibial nerves was higher in diabetic subjects, with ORs of 3.611 for the left common peroneal nerve and 11.077 for the left tibial nerve. Regarding motor conduction, the risk of bilateral slowing was also higher in diabetic subjects, with ORs ranging from 6.3 (right tibial nerve) to 9.758 (right common peroneal nerve) (Table 5).

**Table 5:** Frequency of amplitude and motor conduction abnormalities according to European standards in non-diabetic and diabetic subjects.

Abnormality type	Nerve	Side	All subjects (*n* = 51)	Non-diabetics (*n* = 26)	Diabetics (*n* = 25)	OR	95% CI
Amplitude reduction	EPS motor	R	12 (23.5)	3 (11.5)	9 (36.0)	4.31	1.0–18.5
		L	7 (13.7)	2 (7.7)	5 (20.0)	3.00	0.5–17.1
	EPS sensory	R	22 (43.1)	7 (26.9)	15 (60.0)	4.71	1.2–13.2
		L	19 (37.3)	6 (23.1)	13 (52.0)	3.61	1.1–12.0
	IPS motor	R	20 (39.2)	6 (23.1)	14 (56.0)	4.24	1.3–14.2
		L	21 (41.2)	8 (30.8)	13 (52.0)	2.44	0.8–7.6
	IPS sensory	R	20 (39.2)	6 (23.1)	14 (56.0)	4.24	1.3–14.2
		L	14 (27.5)	2 (7.7)	12 (48.0)	11.08	2.1–57.2
Conduction slowing	EPS motor	R	17 (33.3)	3 (11.5)	14 (56.0)	9.76	2.3–41.1
		L	16 (31.4)	3 (11.5)	13 (52.0)	8.31	1.9–34.9
	IPS motor	R	20 (39.2)	5 (19.2)	15 (60.0)	6.30	1.8–22.2
		L	21 (41.2)	5 (19.2)	16 (64.0)	7.47	2.1–26.6

EPS, external popliteal sciatic nerve (common peroneal nerve); IPS, internal popliteal sciatic nerve (tibial nerve); OR, odds ratio; CI, confidence interval. Data are presented as *n* (%). Values in bold indicate statistically significant associations (95% CI excludes 1.0).

### 3.5 Patient-level analysis

To identify clinical and biological factors associated with the presence of electroneurographic abnormalities in diabetic patients, we performed an analysis comparing diabetic patients with normal versus abnormal ENG findings (Table 6). Patients with abnormal ENG had significantly longer diabetes duration (*p* = 0.04), higher HbA1c levels (*p* = 0.02), and were more likely to have neuropathic pain (DN4 ≥ 4) (*p* = 0.03). Burning sensations (*p* = 0.01) and tingling (*p* = 0.01) were also more frequent in the abnormal ENG group.

**Table 6:** Clinical characteristics of diabetic patients according to electroneurographic (ENG) status.

Characteristic	Normal ENG (*n* = 8)	Abnormal ENG (*n* = 17)	*p*-value*
Age, years (mean ± SD)	52.1 ± 9.8	56.4 ± 10.2	0.34
Diabetes duration, years (median, IQR)	5 (2–10)	10 (5–18)	**0.04**
HbA1c, % (median, IQR)	6.2 (5.6–6.9)	7.1 (6.3–7.8)	**0.02**
Insulin treatment, *n* (%)	2 (25.0)	8 (47.1)	0.31
DN4 ≥ 4, *n* (%)	3 (37.5)	14 (82.4)	**0.03**
Burning sensation, *n* (%)	3 (37.5)	15 (88.2)	**0.01**
Tingling, *n* (%)	3 (37.5)	15 (88.2)	**0.01**
Numbness, *n* (%)	2 (25.0)	11 (64.7)	0.08

ENG, electroneurography; HbA1c, glycated hemoglobin; DN4, Douleur Neuropathique 4 questionnaire. Abnormal ENG was defined as the presence of at least one electrophysiological abnormality (reduced amplitude, prolonged distal latency, or reduced conduction velocity) in any nerve. Values in bold indicate statistically significant differences (*p* < 0.05). Statistical tests: Mann–Whitney test for continuous variables; *χ*^2^ or Fisher’s exact test for categorical variables.

## 4 Discussion

This study aimed to characterize motor and sensory electroneurographic parameters in diabetic subjects compared to non-diabetic subjects, with the goal of contributing to the development of early screening strategies for peripheral neuropathy in the Congolese context. The association of the DN4 score with electroneurographic abnormalities is based on the fact that diabetic peripheral neuropathy (DPN) frequently begins with a painful phase before the appearance of frank clinical signs (26).

### 4.1 Predominant electrophysiological alterations in diabetic patients

The main finding of this study is that diabetic patients in Lubumbashi present significant alterations in nerve conduction parameters compared to non-diabetic controls, particularly in the lower limbs. This finding highlights the existence of diabetic peripheral neuropathy (DPN) in this population and emphasizes the value of electroneurography (ENG) for early screening.

Diabetic subjects showed significant reductions in motor and sensory conduction velocities, decreased amplitudes, and prolonged distal latencies compared to non-diabetic controls (Table 3). These abnormalities were more pronounced in the lower limbs, affecting the common peroneal and tibial nerves. This pattern predominantly axonal, sensory-motor polyneuropathy with demyelinating features Is consistent with the literature (12, 27). The bilateral and symmetric distribution supports the diagnosis of distal symmetric polyneuropathy, the most common form of DPN.

The patient-level analysis confirms that diabetic patients with abnormal ENG have more severe risk factors: longer diabetes duration (*p* = 0.04), higher HbA1c (*p* = 0.02), and more frequent neuropathic pain (*p* = 0.03). These results are consistent with the work of Partanen et al. (14), who established the link between diabetes duration, glycemic control, and neuropathy development.

### 4.2 Neuropathic pain and clinical correlates

Neuropathic pain, assessed by the DN4 questionnaire, was significantly more frequent in diabetic subjects (68%) than in non-diabetic controls (46%). The most reported symptoms were burning sensations, tingling, and numbness. ENG abnormalities were observed in diabetic patients without neuropathic pain, suggesting the presence of subclinical DPN. This finding reinforces the clinical utility of ENG for early diagnosis, even in asymptomatic patients (20, 21).

The higher frequency of itching in diabetic subjects (52% vs. 23.1%, *p* = 0.04) corroborates findings in the literature, which report a prevalence ranging from 18.4 to 35.8% in type 2 diabetic patients (12, 28, 29). These results reinforce the concept of focal and multifocal neuropathy, where pain and electrophysiological abnormalities coexist inconsistently.

### 4.3 Specificity of our population: motor predominance and local factors

In contrast to earlier studies reporting a sensory predominance in diabetic neuropathy (14, 27), our results show a more marked motor involvement. This specificity may be explained by several factors:

1. *Diabetes duration and glycemic control*: Our patients had a median diabetes duration of 10 years (IQR 5–18) and a median HbA1c of 6.85%, suggesting suboptimal glycemic control, which may promote early motor involvement (30–33).
2. *Environmental exposure*: Lubumbashi is a mining region where chronic exposure to heavy metals (lead, cadmium, copper, cobalt) is common. These metals are known for their neurotoxicity, which may aggravate or modify the phenotype of diabetic neuropathy (34–37).
3. *Nutritional and deficiency factors*: In our setting, vitamin and trace element deficiencies (38, 39) could influence the clinical and electrophysiological presentation of neuropathy.

### 4.4 Hypotheses regarding ENG abnormalities in non-diabetic subjects

ENG abnormalities were observed in 46% of non-diabetic subjects. While this finding is notable, we did not perform heavy metal assays. We therefore hypothesize that environmental factors particularly chronic exposure to heavy metals from mining activities in Lubumbashi may contribute to these abnormalities (35). However, other potential causes should also be considered, including:

- Chronic alcoholism
- Nutritional deficiencies (vitamin B12, folate)
- Chronic infections (HIV, tuberculosis, leprosy)
- Drug-induced neurotoxicity (antiretrovirals, isoniazid)
- Endocrine or metabolic disorders (hypothyroidism, renal failure)

These hypotheses should be explored in future studies with appropriate biological assays and comprehensive clinical evaluation.

### 4.5 Clinical implications and future directions

The abnormalities in conduction amplitudes and velocities observed even in subjects without neuropathic pain highlight the value of electroneurography (ENG) for early diagnosis. ENG enables the identification of subclinical forms of neuropathy, often missed by symptomatic evaluation alone (20, 21).

The prolongation of distal latencies, decrease in motor amplitudes, and reduction in conduction velocities observed in diabetic subjects indicate predominantly demyelinating motor involvement, sometimes associated with a multifocal axonal component. This motor predominance contrasts with some studies that report primarily sensory involvement in DPN, which may reflect the heterogeneous and progressive nature of diabetic neuropathic forms (12).

The systematic integration of ENG into the follow-up of diabetic patients, combined with clinical screening tools such as the DN4, therefore appears essential to improve early diagnosis and provide early treatment, sparing patients from the dreaded complications of the disease.

### 4.6 Strengths and limitations of the study

#### 4.6.1 Strengths

Despite its limitations, this study has several strengths. It is one of the first studies in Lubumbashi to combine clinical assessment of neuropathic pain using a validated tool (DN4) with objective electroneurographic measurements in diabetic subjects. The inclusion of a non-diabetic comparison group from the same environment allows differentiation of diabetes-related abnormalities from those potentially caused by other local exposures.

*Limitations*: This study has several limitations that should be acknowledged:

1. *Sample size*: Longitudinal studies and/or studies with larger samples are needed to confirm our findings.
2. *Lack of local norms*: The absence of electroneurographic reference values specific to the Congolese population makes it difficult to precisely interpret observed variations.
3. *Lack of heavy metal assays*: The absence of biological measurements of heavy metals limits our ability to confirm or refute the environmental hypothesis.

## 5 Conclusion

This study demonstrates that diabetic patients in Lubumbashi have significantly altered electroneurographic parameters (amplitude, distal latency, and conduction velocity) compared to non-diabetic controls, with a predominance of axonal, sensory-motor polyneuropathy affecting the lower limbs. Neuropathic pain was more frequent and severe in diabetic patients, particularly in those with insulin dependence. The combination of ENG with the DN4 questionnaire improves the detection of both overt and subclinical neuropathy. Patient-level analysis showed that abnormal ENG is associated with longer diabetes duration, poor glycemic control, and increased prevalence of neuropathic pain. The presence of ENG abnormalities in non-diabetic subjects raises the hypothesis of environmental or other non-diabetic causes of neuropathy in this region, which should be investigated in future studies. Integrating ENG into routine clinical practice is essential for early diagnosis and prevention of diabetic neuropathy complications. Further studies with larger samples, comprehensive environmental exposure assessment, and local normative data are needed to refine these findings.

## Data Availability

The data analyzed in this study is subject to the following licenses/restrictions: We told the participants that the data would remain confidentials. Requests to access these datasets should be directed to Jean-Paul Ngoy Mande, jp.ngoymande@gmail.com.
